# A New Augmentation Method for Improved Screw Fixation in Fragile Bone

**DOI:** 10.3389/fbioe.2022.816250

**Published:** 2022-03-02

**Authors:** Deepak Bushan Raina, Vetra Markevičiūtė, Mindaugas Stravinskas, Joeri Kok, Ida Jacobson, Yang Liu, Erdem Aras Sezgin, Hanna Isaksson, Stefan Zwingenberger, Magnus Tägil, Šarūnas Tarasevičius, Lars Lidgren

**Affiliations:** ^1^ Department of Clinical Sciences Lund, Orthopedics, The Faculty of Medicine, Lund University, Lund, Sweden; ^2^ Department of Orthopedics and Traumatology, Lithuanian University of Health Sciences, Kaunas, Lithuania; ^3^ Department of Biomedical Engineering, Lund University, Lund, Sweden; ^4^ Department of Orthopedics and Traumatology, Faculty of Medicine, Aksaray University, Aksaray, Turkey; ^5^ University Hospital Carl Gustav Carus at Technische Universität Dresden, University Center of Orthopedic, Trauma and Plastic Surgery, Dresden, Germany

**Keywords:** biomaterial, osteoporosis, hip fracture, implant integration, implant augmentation

## Abstract

Pertrochanteric fractures (TF) due to osteoporosis constitute nearly half of all proximal femur fractures. TFs are treated with a surgical approach and fracture fixation is achieved using metallic fixation devices. Poor quality cancellous bone in osteoporotic patients makes anchorage of a fixation device challenging, which can lead to failure of the fracture fixation. Methods to reinforce the bone-implant interface using bone cement (PMMA) and other calcium phosphate cements in TFs have been described earlier but a clear evidence on the advantage of using such biomaterials for augmentation is weak. Furthermore, there is no standardized technique for delivering these biomaterials at the bone-implant interface. In this study, we firstly describe a method to deliver a calcium sulphate/hydroxyapatite (CaS/HA) based biomaterial for the augmentation of a lag-screw commonly used for TF fixation. We then used an osteoporotic Sawbones model to study the consequence of CaS/HA augmentation on the immediate mechanical anchorage of the lag-screw to osteoporotic bone. Finally, as a proof-of-concept, the method of delivering the CaS/HA biomaterial at the bone-implant interface as well as spreading of the CaS/HA material at this interface was tested in patients undergoing treatment for TF as well as in donated femoral heads. The mechanical testing results indicated that the CaS/HA based biomaterial increased the peak extraction force of the lag-screw by 4 times compared with un-augmented lag-screws and the results were at par with PMMA. The X-ray images from the patient series showed that it was possible to inject the CaS/HA material at the bone-implant interface without applying additional pressure and the CaS/HA material spreading was observed at the interface of the lag-screw threads and the bone. Finally, the spreading of the CaS/HA material was also verified on donated femoral heads and micro-CT imaging indicated that the entire length of the lag-screw threads was covered with the CaS/HA biomaterial. In conclusion, we present a novel method for augmenting a lag-screw in TFs, which could potentially reduce the risk of fracture fixation failure and reoperation in fragile osteoporotic patients.

## Introduction

More than 200 million individuals are suffering from osteoporosis and the number is expected to double in 2050 (Odén et al., 2015). A common manifestation of the disease is a pronounced reduction in the bone mineral density (BMD) and an increase in porosity of trabecular bone (NIH Concensus development panel, 2001). This makes anchorage of fracture fixation devices such as screws challenging in fragile cancellous bone.

It is estimated that nearly 2.5 million individuals sustain a proximal femur fracture yearly and the number is expected to reach 5 million in the year 2050 (Gullberg et al., 1997; Odén et al., 2015). Pertrochanteric femur fractures (TF), which account for half of all proximal femur fractures (Grimsrud et al., 2005), are often surgically treated using either an intramedullary nail with a sliding hip screw or blade. Alternatively, a lateral plate is used in combination with a sliding screw. Failures due to loss of fixation in the femoral head is a prominent risk attributed to these internal fixation methods, mostly caused by inferior quality of the cancellous bone around the screw or insufficient reposition. Rate of failure due to cut-out of the femoral head that requires revision surgery is about 3% for both methods. In addition, around 2% of the cases are reported to have a postoperative infection (Edwards et al., 2008). The proximal femur fracture patients in general are fragile and the 1-year mortality in patients >60 years of age ranges between 10 and 40%, predominantly determined by age and comorbidities (Mattisson et al., 2018). An additional re-operation in an already fragile patient, reduces quality of life and most importantly, increases the risk of premature death.

In order to improve the mechanical anchorage of the screw and prevent re-operations, various attempts have been made to reinforce the fragile bone with polymer based injectable materials. Cadaver studies and small sized clinical studies have indicated a preventive effect of poly methyl methacrylate (PMMA) based augmentation on screw or blade cut-out but evidence from larger randomized controlled trials is lacking (Stoffel et al., 2008; Gupta et al., 2012). Drawbacks include the exothermic setting reaction of PMMA, which can lead to necrosis of the femoral head, and the inability of the material to remodel into living bone. These drawbacks have garnered skepticism and thus slow clinical acceptance (Whitehouse et al., 2014). Calcium phosphate (CaP) cements in small clinical trials have also been proven to be effective in improving fracture stability but again no strong evidence from larger clinical trials (Mattsson and Larsson, 2004; Fuchs et al., 2019).

Furthermore, there appears to be a genuine lack of commonly acknowledged consistent surgical methods for the different augmentation procedures. Previous studies have used pre-filling of the entire reamed canal in the femoral head with PMMA (Gupta et al., 2012; Rai et al., 2018). Limitations with this method are an increased risk of the excessive PMMA spreading into the venous system and osteonecrosis as a result of the exothermic setting reaction. Others have injected isothermally setting CaP cement into the entire reamed lag-screw canal, and in another smaller canal parallel to the reamed lag-screw canal (Mattsson and Larsson, 2006). Injections through fenestrated lag-screws have also been tested (Mattsson and Larsson, 2004; Stoffel et al., 2008; Fuchs et al., 2019). Although the concept of using biodegradable materials is compelling, several limitations need to be addressed for standardizing the surgical technique; 1) exactly controlling the volume and spreading of the delivered material, 2) the time constraint wherein the surgeon needs to fully insert the lag-screw before the material sets and, 3) using a delivery technique avoiding leakage into the venous system around the femoral head and neck. These limitations pose significant challenges for the standardization of material delivery in the augmentation of screws in fragile bone.

Herein, we report the augmentation of a lag-screw using an isothermally setting bioresorbable calcium sulphate/hydroxyapatite (CaS/HA) biomaterial in an operating room setting utilizing fluoroscopy and standard surgical equipment. The CaS/HA material is a powder-based biomaterial which upon mixing with a radiopaque contrast agent forms a paste which is injectable for approximately 5 min after mixing, moldable up to 9 min post mixing and sets into a solid mass at approximately 15 min from the start of the mixing procedure (Abramo et al., 2010). The compressive strength of the material is higher than cancellous bone (Nilsson et al., 2003). A recent study has verified that the CaS/HA material spreads in the trabecular structures and protects the bone from fracturing at low loads compared to control trabecular bone (Kok et al., 2021b).

We hypothesized that by standardizing the sequence of steps during TF surgery and by controlling the delivery of a CaS/HA material introduced at the interface of the lag-screw and osteoporotic bone, it would be possible to enhance the immediate anchorage of the lag-screw to bone. To verify the immediate increase in the anchorage of the lag-screws to osteoporotic bone, an osteoporotic bone analog in the form of Sawbones was used for mechanical testing. The lag-screws were left un-augmented, augmented with the CaS/HA material or PMMA as a control. We further aimed to illustrate the intraoperative technique for augmenting lag-screws during a routine TF fixation procedure.

## Materials and Methods

### Study Design

In this study, we first tested the immediate mechanical anchorage of a lag-screw to Sawbones (bone analog replicating osteoporotic bone) using PMMA and CaS/HA biomaterials at the screw-bone interface. We then reported the surgical technique and the method to deliver the CaS/HA biomaterial at the lag-screw-bone interface in patients undergoing surgical treatment for fixation of an inter-trochanteric fracture. Finally, we evaluated the spreading of the CaS/HA biomaterial at the lag-screw-bone interface in donated femoral heads from osteoporotic patients undergoing a total hip arthroplasty.

### Lag-Screw Anchorage in Osteoporotic Sawbones Blocks

**FIGURE 1:**
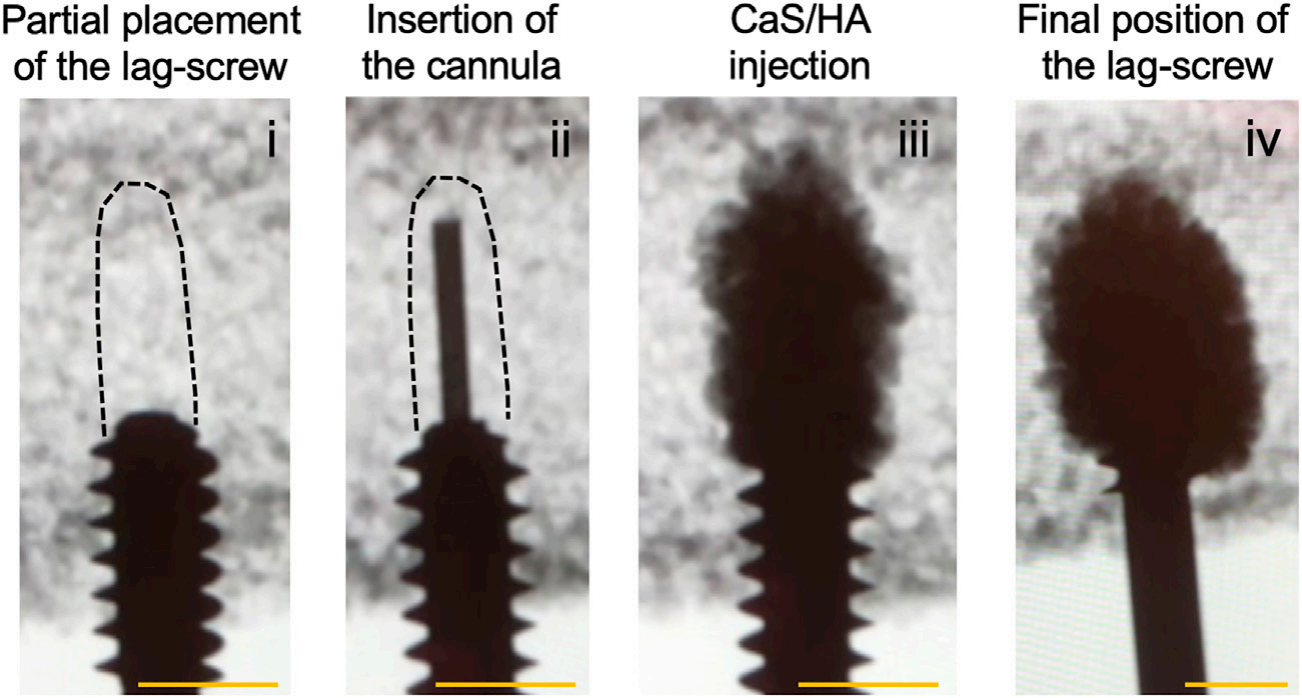
CaS/HA biomaterial-based augmentation of a lag-screw in an osteoporotic Sawbones model. An overview of experimental steps is provided in the radiological time-lapse images starting from (i) the partial placement of the lag-screw followed by (ii) insertion of the injection device through the lag-screw (ii). (iii) CaS/HA biomaterial filled in the pre-drilled canal and (iv) the final position of the lag-screw surrounded by the CaS/HA material. Dashed black lines in (i) and (ii) indicate the approximate position of the pre-drilled region. Scale bar = approximately 1.3 cm.

Open-cell, rigid foam Sawbones blocks (SAWBONES Europe AB, Sweden) with a 15% bone volume fraction (BV/TV) (Catalog number: #1522-524) were used to mimic the open porous structure of human osteoporotic bone. Each block measuring 5 cm × 6 cm x 4 cm (l x b x h) was predrilled with an 8 mm burr to a depth of 3 cm. At this point, a stainless-steel lag-screw (Auxein Medical Pvt. Ltd., India) with a length of 11.5 cm, 1.25 cm thread diameter and 0.8 cm shaft diameter was partially inserted into the pre-drilled hole to an approximate depth of 0.5 cm (Figure 1).

The CaS/HA biomaterial was mixed with a radiopaque contrast agent following the manufacturer’s guidelines (Bonesupport AB, Sweden) and mixed for 30 s. The CaS/HA paste was then transferred into a 5-cc injection syringe. A custom-made titanium cannula (h = 16 cm, diameter = 2.5 mm) with a luer lock adapter was connected to the syringe containing the pre-mixed CaS/HA material. At t = 2.5 min after start of the mixing, the titanium cannula was inserted in the Sawbones block through the cannulated lag-screw and placed at the most distal end of the pre-drilled canal (Figure 1(ii)). The material was then gradually injected while slowly retracting the cannula until the entire pre-drilled canal and the surrounding bone was filled with 2 ml of the CaS/HA paste (Figure 1(iii)). The lag-screw was then immediately advanced forward to its final intended position (Figure 1(iv)). This procedure was carried out on n = 9 specimens. Similar procedure was repeated for samples containing 2 ml of low-viscosity PMMA (Osteopal^®^ Low Viscosity, Biomet Merck, Switzerland) (n = 7) and the injection was performed after a 5 min waiting time post mixing. Lag-screws without biomaterial augmentation were used as controls (n = 8). After a setting period of 24 h, the lag-screws were pulled out using a hydraulic mechanical testing machine (Instron^®^ 8511.20, U.S.A) and the data was recorded on a digital acquisition system MTS FlexTest 40 controller (MTS TestSuite Multipurpose Elite Software). A pre-established tension protocol was used to test the pull-out strength of the augmented lag-screws (Kok et al., 2021a). Briefly, the saw bone blocks with the attached lag-screws were placed in a cuboidal metal frame with an opening on the top through which the lag-screw was passed. The metal frame was attached to an adjustable clamp while the compression screw canal in the lag-screw was used to insert a metal hook, which was connected to the load sensor. Specimens were then pulled at a displacement rate of 0.5 mm/s until failure and the force-displacement curve was used to calculate the peak failure force, stiffness and work.

### Spreading of the CaS/HA Biomaterial (Ex-Vivo)

Femoral heads (n = 2) from osteoporotic patients undergoing hip replacement following a low-energy cervical neck fracture were obtained from the tissue bank at Kaunas University Hospital. Tissue banking was performed after approval from the Institute Review Board at the Kaunas University Hospital and by following the EU legislation on tissue banking. Informed patient consent was obtained to use the femoral heads for research. Fresh frozen femoral heads were thawed overnight at 4 °C a day prior to augmenting the lag-screw-bone interface with the CaS/HA biomaterial. On the day of the procedure, an 8 mm burr was used to drill a 3 cm long hole in the femoral head under fluoroscopic guidance. A lag-screw (11.5 cm long) was partially inserted into the pre-drilled hole up to an approximate distance of 0.5 cm. At this point, CaS/HA material was used to fill the canal in front of the screw with 2.5 ml material. Finally, the screw was fastened and placed at its final intended position as verified by fluoroscopy. The augmented femoral head was imaged with a micro-CT scanner (MI labs, Netherlands) to evaluate the distribution of the CaS/HA material around the screw-bone interface. After the completion of the CT, the bone was left at -20°C overnight, after which the lag-screw was manually removed from the bone. Care was taken to minimize disturbance to the interface region or the cancellous bone by performing the procedure on frozen bone. The bone without the lag-screw was then thawed again and photographs of the femoral heads were obtained to confirm the presence of the CaS/HA biomaterial at the bone-lag-screw interface.

### Surgical Method for CaS/HA Augmentation of the Lag-Screw During TF Treatment

**FIGURE 2:**
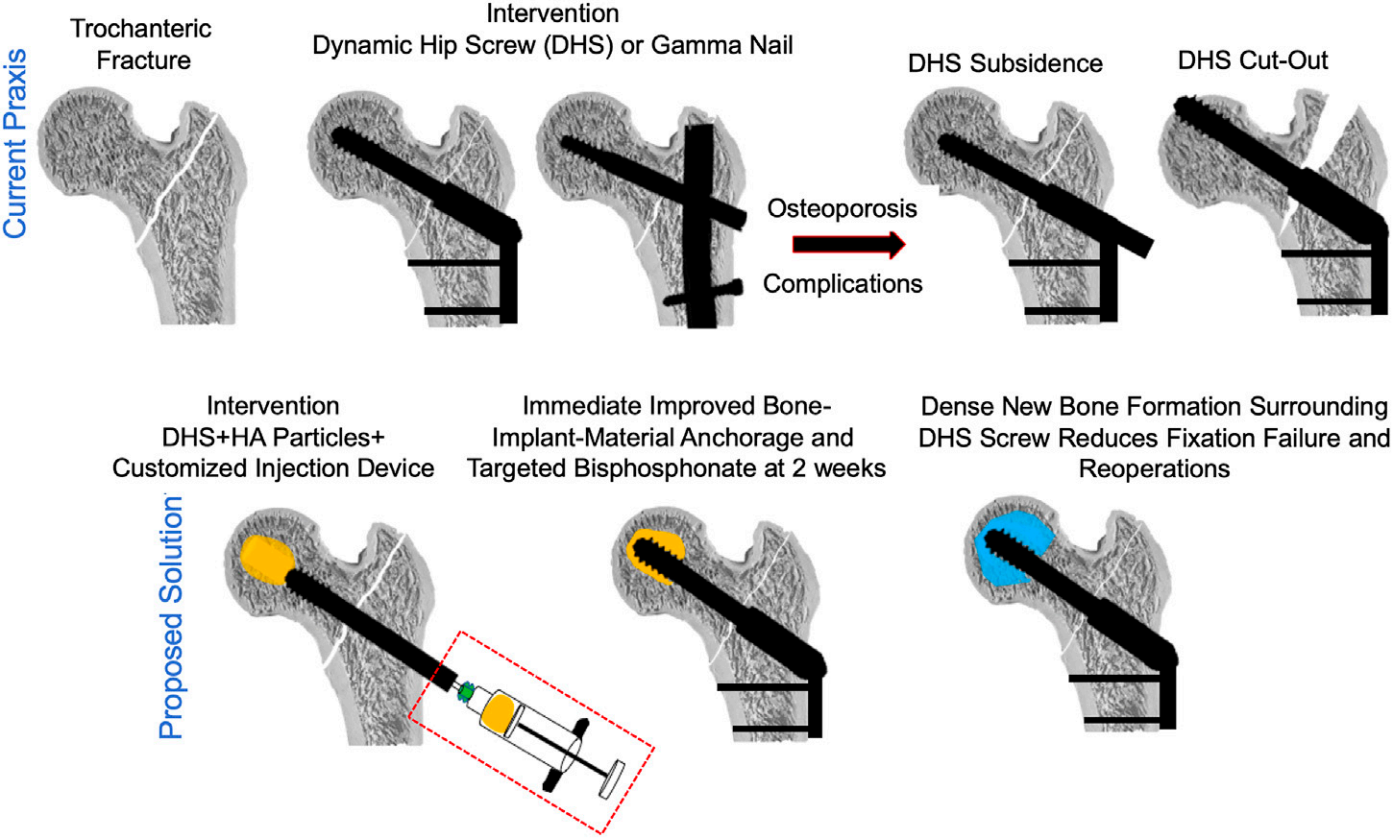
A schematic indicating the current praxis used for the surgical management of pertrochanteric fractures (top) and the proposed solution of augmenting the lag-screw with a CaS/HA biomaterial to reduce fracture fixation device failure (bottom).

A schematic of the current praxis of trochanteric fracture fixation, possible complications with fracture fixation as well as the proposed augmentation method using the CaS/HA biomaterial at the lag-screw-bone interface are shown in Figure 2 and Video 1.

**FIGURE 3:**
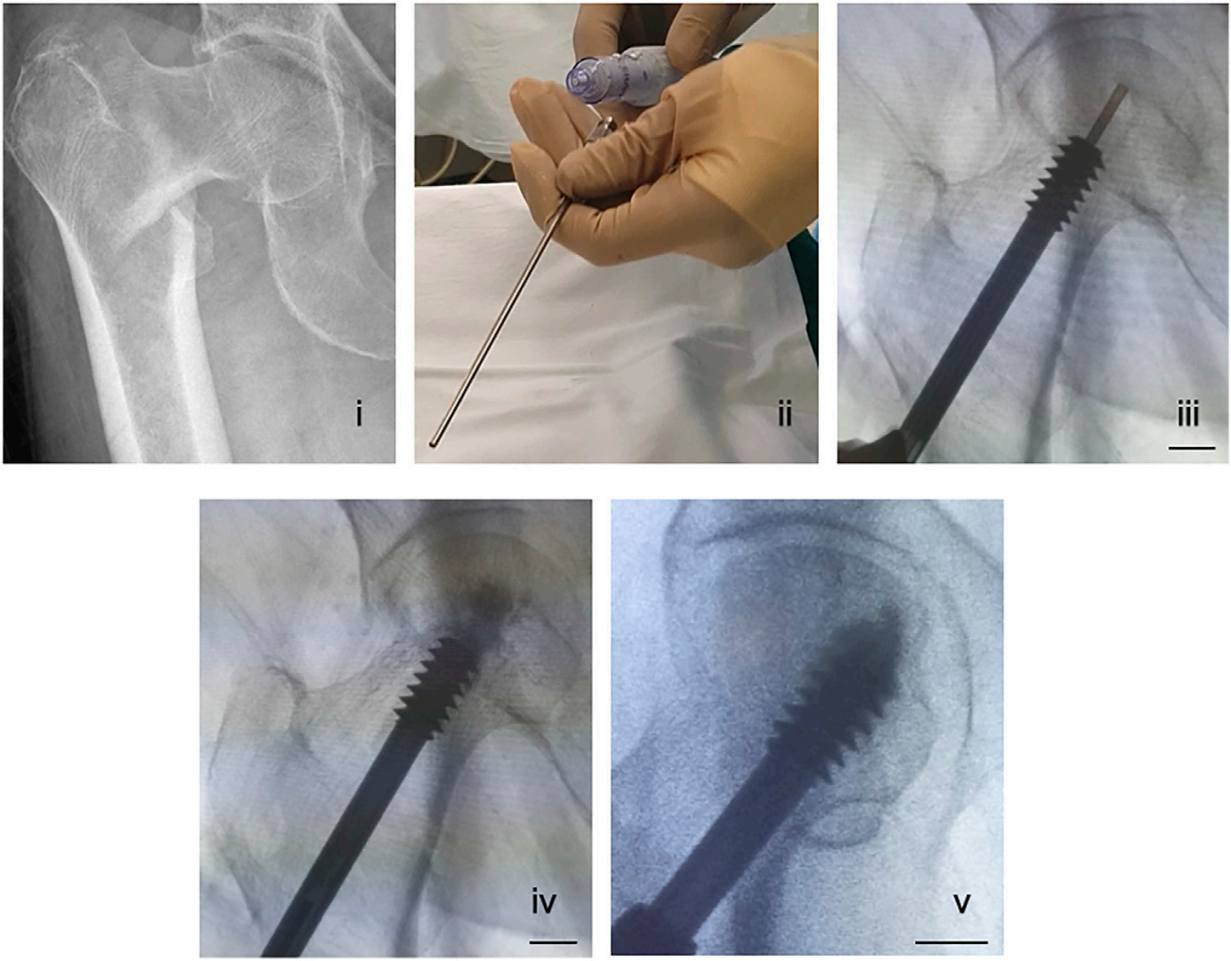
A surgical method of delivering a CaS/HA biomaterial through a cannulated lag-screw in a patient undergoing treatment for pertrochanteric fracture. (i) X-ray showing a pertrochanteric fracture. (ii) A photograph of the injection cannula being connected to a regular injection syringe containing the CaS/HA biomaterial. (iii) Placement of the titanium cannula at the most distal end of the pre-drilled canal via the cannulated lag-screw visualized radiographically. (iv) Radiographic image of the CaS/HA biomaterial being injected into the pre-drilled canal in-front of the lag-screw. (v) Radiograph of the final position of the lag-screw with the biomaterial around. Scale bar in panels iii-v is ∼1.3 cm.

To test the biomaterial delivery at the lag-screw bone interface as well as optimize the surgical procedure in TF patients, a clinical trial of lag-screw augmentation in TFs was approved by the Institute Review Board (IRB) at the Kaunas University Hospital, Kaunas, Lithuania (Ethical permit number: P1 BE-2-76/2019) and the study details can be found at ClinicalTrials.gov (Identifier: NCT04498715). Patients undergoing treatment for trochanteric fracture (Figure 3(i)) with a sliding hip screw and a femoral plate were stratified based on the FAME index and patients with high fracture risk and low-mortality were included in the study (Sezgin et al., 2020). As of today, a total of 5 patients have been included. Patient details are presented in Table 1.

**TABLE 1:** Demographic information of patients included in the TF study.

Patient ID	Age	Gender	Fracture type (AO Classification)
1	95	F	A1
2	80	F	A1
3	84	F	A1
4	72	F	A2
5	86	F	A1

All patients were above the age of 60 who suffered a low energy trauma to the hip. At the time of surgery, a guide wire was inserted into the femoral head until it reached the final intended position of the lag-screw. An 8 mm burr placed over the guide wire was then used to prepare a pilot hole for lag-screw placement. Once the drilling was complete, a lag-screw (Auxein Medical Pvt. Ltd., India) was placed over the guide wire and inserted into the bone 2.5 cm from its final intended position. At this point, an injectable CaS/HA biomaterial was mixed as per the manufacturers guidelines and the paste was transferred into a 10-cc injection syringe. A long titanium cannula with a luer lock was then connected to the syringe (Figure 3(ii)).

The guide wire was removed from the cannulated lag-screw and the cannula connected to the injection syringe was placed into the cannulated lag-screw (Figure 3(iii), Video 2). The CaS/HA biomaterial was injected with fluoroscopic guidance at t = 2.5 min from the start of mixing. Injection was started at the end closer to the joint and the cannula was slowly retracted towards the tip of the lag-screw until the drilled space in front of the lag-screw and the surrounding cancellous bone were filled with 2.5 ml of the material (Figure 3(iv)). Finally, the lag-screw was inserted to its final position and the femoral plate was attached to the lag-screw (Figure 3(v)). Post-operative X-rays were obtained for all patients and the follow up regimen consists of bilateral CT scans at 3 months, CT-scan and DEXA at 6-months and final radiological evaluation using X-rays at 1-year post operation.

### Statistical Analysis

Distribution of the data from the mechanical testing experiment was tested using Shapiro-Wilk normality test. Normally distributed data were analyzed with a one-way ANOVA with Dunnett’s post hoc test for comparison of the test groups with the control group. Kruskal–Wallis test with Dunn’s post hoc method was used for the analysis of non-parametrically distributed data. Statistical significance was set at *p* < 0.05. All statistical tests were conducted on Prism v9 (GraphPad, U.S.A).

## Results

### Lag-Screw Anchorage in Osteoporotic Sawbones Blocks

**FIGURE 4:**
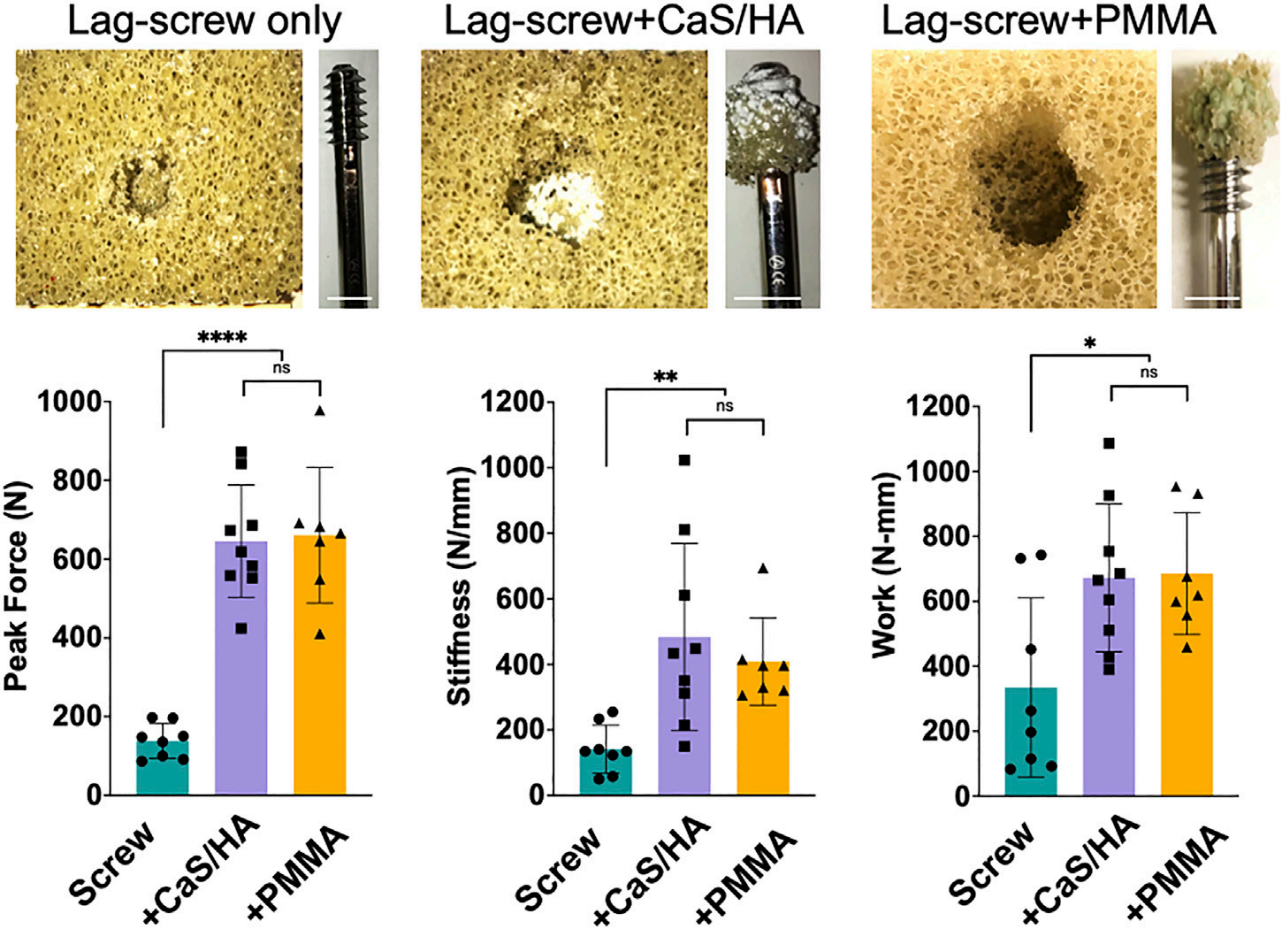
Mechanical effects of CaS/HA augmentation on lag-screw anchorage in Sawbones (Top) Shows photographs of the Sawbones block and the lag-screw after the pull-out test. Notice the spreading of the CaS/HA material in the pre-drilled hole and the presence of both CaS/HA and PMMA biomaterial-Sawbones composite on the lag-screws after pull-out testing (Bottom) Scatter plots showing mean peak force to pull-out the screw, the stiffness and work required until the failure of the screws in the Sawbones model. * Indicates *p* < 0.05, ** indicates *p* < 0.01 and **** indicates *p* < 0.0001. ns indicates not significant. Scale bar indicates 1.3 cm.

Without augmentation, the lag-screw was pulled straight out of the Sawbones blocks without any remnants of the Sawbones being attached to the lag-screw after the pull-out was complete (Figure 4). In the case of both CaS/HA and PMMA, the biomaterials protected the Sawbones from failing at the immediate interface of the lag-screw. The bone analog instead failed at regions where the material was absent (Figure 4).

The peak force (*p* < 0.0001), stiffness (*p* < 0.01) and work (*p* < 0.05) in CaS/HA and PMMA augmented lag-screws was significantly higher than the un-augmented lag-screw (Figure 4). No difference was observed in any of the measured parameters between the CaS/HA and the PMMA augmented lag-screw. It was also noted that the CaS/HA material covered the entire threaded region of the lag-screw while the PMMA material was concentrated mostly at the lower threads.

### Spreading of the CaS/HA Biomaterial (Ex-Vivo)

**FIGURE 5:**
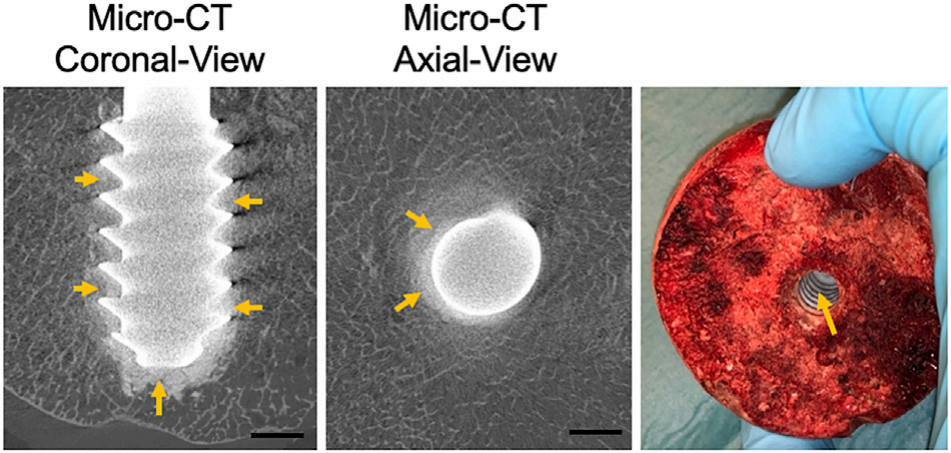
CaS/HA spreading around the lag-screw and bone interface in cadaver femoral head as observed by micro-CT and a digital photograph taken after removal of the lag screw. Scale bar indicates ∼0.5 cm.

The spreading of the CaS/HA biomaterial in the cadaver femoral head was similar to the sawbones specimens. Micro-CT images clearly demonstrated that the CaS/HA material occupied the space between the lag-screw threads as well as in the surrounding bone (Figure 5). Presence of CaS/HA material was also confirmed after removal of the lag-screw from the femoral head (Figure 5).

### Surgical Method for CaS/HA Augmentation of the Lag-Screw During TF Treatment

**FIGURE 6:**
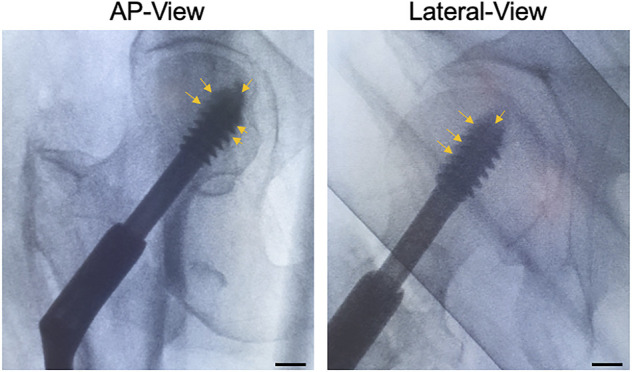
CaS/HA spreading around the lag-screw in patients undergoing pertrochanteric fracture fixation using a sliding lag-screw and a femoral plate. Arrows indicate the radio dense CaS/HA material around the lag-screw-bone interface. Scale bar indicates ∼1.3 cm.

Once the lag-screw was placed at its final intended position, radiopaque CaS/HA material was observed at the lag-screw and bone interface as well as the tip of the lag-screw in both AP and lateral views (Figure 6).

## Discussion

In this study, we described a novel method of biomaterial delivery for augmentation of a lag-screw used in the treatment of TFs. Instead of injecting the CaS/HA material through a cannulated device or completely filling the pilot hole, we used the pilot hole to partially insert the lag-screw into the bone. The void in front of the pilot hole was then filled by placing an instrument placed in the cannulation of the lag-screw to deliver a limited volume of a self-setting biomaterial at a pre-drilled space in front of a lag-screw. By using this approach, the biomaterial was distributed uniformly at the interface of the lag-screw threads and the surrounding bone without the use of high pressure or the risk of leakage in surrounding venous structures. We also demonstrated that the described method delivering an injectable CaS/HA biomaterial improved the immediate anchorage of the lag-screw with a >4-fold increase in the extraction force in a sawbones model mimicking osteoporotic bone. The increase in the mechanical integration of the lag-screw was similar to the current clinical standard material, PMMA. These results are in agreement with a recent meta-analysis wherein the authors evaluated the biomechanical effects of lag-screw augmentation using calcium phosphate cement or PMMA in 40 different studies and concluded that cement augmentation of lag-screws for the treatment of TFs improves early screw anchorage both *ex-vivo* and in small sized clinical studies (Goodnough et al., 2021). Although immediate anchorage of the implant is of importance for early fixation, it would be a breakthrough if biomaterial-based augmentation also promoted new bone formation both around the lag-screw as well as in the trochanteric fragility fracture void. A study by Wu and co-workers indicated that despite the biomechanical advantages achieved from PMMA augmentation of lag-screws in prevention of screw cut-out, delayed union or nonunion in the TFs was observed in 9% of the cases (Wu et al., 2012). A similar result was reported by Mattsson and co-workers in their study involving the use of a calcium phosphate cement used for augmentation of screws for displaced femoral neck fractures (Mattsson and Larsson, 2006). In a case series, Mindaugas and co-workers demonstrated that by injecting the CaS/HA material in the fracture gap in patients undergoing treatment for TF with lag-screw fixation, all patients healed with minimal subsidence of the lag-screw (Stravinskas et al., 2018). In the same study, histological specimens from one of the patients that required re-operation due to hardware failure unrelated to the augmentation procedure demonstrated viable bone formation around the lag screw as well as near the fracture.

CaS/HA is a composite of calcium sulphate dihydrate and hydroxyapatite. The CaS phase, which forms the majority of the biomaterial (60% by weight), undergoes dissolution in body fluids in about 6-weeks *in vivo* (Wang et al., 2016). This is contrary to the degradation rate of calcium phosphate based cements, which may take years to resorb (Young et al., 1998). Owing to a balanced material degradation-osteoconduction rate of the CaS/HA biomaterial, it has previously been demonstrated that the biomaterial can promote efficient bone healing in clinical scenarios (Abramo et al., 2010; Zampelis et al., 2013). Furthermore, animal studies have demonstrated that the resorbed CaS phase is replaced by new bone as early as 4-weeks (Raina et al., 2016). However, the bone induction in challenging fractures and critical bone defects cannot be achieved by the CaS/HA biomaterial alone (Raina DB. et al., 2020). This is where the ability of the CaS/HA biomaterial to be used also as a carrier for bioactive molecules is pivotal. Preclinical studies have indicated that the biphasic material can be used as an efficient local carrier for bone forming agents such as bone morphogenic protein-2 (BMP-2) and bisphosphonates in challenging models of bone healing, but regulatory approvals are needed before these methods become a clinical reality (McNally et al., 2016; Raina et al., 2016; Raina et al., 2019; Raina DB. et al., 2020; Dvorzhinskiy et al., 2021). However, by exploring the chemical affinity of bisphosphonates such as zoledronic acid (ZA) to HA, both of which are approved by the food and drug administration (FDA), our group recently reported an entirely novel concept of enhancing peri-implant bone formation and consequent anchorage (Raina D. B. et al., 2020). It was demonstrated that by implanting a hollow implant containing millions of CaS/HA particles in and around the implant, it was possible to send ZA to the implant via the blood stream, a concept christened as *biomodulation*. Once bound to HA in the CaS/HA material, the HA particles were biomodulated by the ZA and a significantly higher amount of bone formation was observed around the implant using micro-CT, histology and mechanical testing (Raina D. B. et al., 2020). This idea is currently being verified in a small clinical feasibility trial in patients undergoing treatment of TFs with a lag-screw and a femoral plate (ClinicalTrials.gov Identifier: NCT04498715). The bone quality around the lag-screw measured with BMD and CT is the primary outcome variable of the study with screw subsidence and cut-out as the secondary outcome variables.

Apart from the late biological consequences of augmenting the lag-screw, the initial mechanical anchorage of screws in osteoporotic bone is of significance. Aprato et al., in their clinical study comparing the association between early mobility after a proximal femur fracture and mortality reported that there was a statistically significant correlation between early mobilization (as early as 10 days after trauma) and mortality (Aprato et al., 2020). In another earlier experimental study, Kok and co-workers used a similar osteoporotic Sawbones model to evaluate the anchorage of a lag-screw to Sawbones blocks using regular and fenestrated lag-screws with CaS/HA biomaterial delivered using a regular 18G injection needle (Kok et al., 2021a). Lag-screws without CaS/HA material were used as controls. In that study, we demonstrated that when the regular lag-screw was augmented with the CaS/HA biomaterial, a 46% increase in the peak pull-out force could be observed compared with the control lag-screws. This increase in peak force was further substantiated by the use of fenestrated lag-screws (103% increase in peak extraction force). Contrary to the study by Kok et al., in this study, we used a specially designed device to deliver the CaS/HA biomaterial through the lag-screw enabling the material to be delivered precisely in the pre-drilled canal in a controlled fashion. Furthermore, by controlling the bore size and the length of the cannula, we ensured that the CaS/HA material does not flow backwards and leak from the proximal end of the lag-screw. By modifying the biomaterial delivery technique using a specialized delivery device, which increased the CaS/HA biomaterial spreading at the screw-bone interface, we now report that the extraction force of the lag-screw augmented with the CaS/HA biomaterial increased by >350% in the same experimental setup, which is a substantial increase compared with the previous report. Furthermore, by using the new delivery method, standard lag screws without additional fenestrations can be used, which reduces the hardware costs. It is also essential to mention that this augmentation procedure is not only limited to treatment of TFs. Biomaterial based augmentation has also been reported in other anatomical locations with fragile bone and screw fixation in the spine, knee and shoulder (Leichtle et al., 2016; Aneja et al., 2021).

Although, the described method of lag-screw augmentation is in its early stages, important validation studies are currently being conducted in patients undergoing treatment for TFs. We do foresee some possible limitations of this method. First and foremost, contrary to PMMA, the CaS/HA material is a resorbing biomaterial. Despite an increase in immediate screw anchorage as shown in our bone analog Sawbones study, the instrument and the method remain to be tested in human femoral heads. *In-vivo* biomechanical and biological conditions could be more dynamic, and the material resorption rate would require to match that of the bone ingrowth in-order to provide optimal outcome. Secondly, for the concept of biomodulation to work and exert a bone regenerating effect around the HA particles, sufficient recipient HA particles must be exposed to receive the ZA. Although, 2–3 ml of the CaS/HA material has been demonstrated to be sufficient to enhance the immediate anchorage of the lag-screw to the surrounding bone, whether this volume of the material also is sufficient to give an optimal bone regeneration around the screw remains to be proven. Finally, this study describes augmentation of a single type of fixation device (dynamic hip screw system) for the treatment of TFs and whether the results hold true for other fixation devices (such as the intramedullary nailing system or proximal femoral screws) needs to be tested before clinical translation.

## Conclusion

In this study, we describe a novel method of augmenting a lag-screw by delivering a CaS/HA biomaterial at the interface of low-density osteoporotic bone and the screw threads. By means of an isothermic setting reaction, the CaS/HA biomaterial sets into a solid mass and enhances the lag-screw anchorage to the Sawbones based bone analog model. CaS/HA augmented lag-screws demonstrated significantly higher (4-times) extraction forces compared with the un-augmented samples while the extraction force of the CaS/HA augmented lag-screws was at par with PMMA augmented lag-screws. The developed method of lag-screw augmentation using the CaS/HA biomaterial was validated in cadaver femoral heads as well as in patients undergoing treatment for pertrochanteric fractures. The CaS/HA biomaterial was successfully delivered at the lag-screw-bone interface without the use of high pressure and presence of the injected ceramic material was verified around the screw threads. It is envisaged that the developed screw augmentation method could reduce the complications associated with treatment of TFs.

## Data Availability

The original contributions presented in the study are included in the article/Supplementary Material, further inquiries can be directed to the corresponding author.
